# Development of a Canine Rigid Body Musculoskeletal Computer Model to Evaluate Gait

**DOI:** 10.3389/fbioe.2020.00150

**Published:** 2020-03-11

**Authors:** Nathan P. Brown, Gina E. Bertocci, Gregory J. R. States, Gwendolyn J. Levine, Jonathan M. Levine, Dena R. Howland

**Affiliations:** ^1^Canine Rehabilitation and Biomechanics Laboratory, Department of Bioengineering, J.B. Speed School of Engineering, University of Louisville, Louisville, KY, United States; ^2^Department of Veterinary Pathobiology, College of Veterinary Medicine & Biomedical Sciences, Texas A&M University, College Station, TX, United States; ^3^Department of Small Animal Clinical Sciences, Veterinary Medical Teaching Hospital, Texas A&M University, College Station, TX, United States; ^4^Kentucky Spinal Cord Injury Research Center, University of Louisville, Louisville, KY, United States; ^5^Department of Neurological Surgery, School of Medicine, University of Louisville, Louisville, KY, United States; ^6^Research Service, Robley Rex VA Medical Center, Louisville, KY, United States

**Keywords:** canine, gait, pelvic limb, computer model, biomechanics, muscle activation, muscle force, kinematics

## Abstract

**Background:**

Kinematic and kinetic analysis have been used to gain an understanding of canine movement and joint loading during gait. By non-invasively predicting muscle activation patterns and forces during gait, musculoskeletal models can further our understanding of normal variability and muscle activation patterns and force profiles characteristic of gait.

**Methods:**

Pelvic limb kinematics and kinetics were measured for a 2 year old healthy female Dachshund (5.4 kg) during gait using 3-D motion capture and force platforms. A computed tomography scan was conducted to acquire pelvis and pelvic limb morphology. Using the OpenSim modeling platform, a bilateral pelvic limb subject-specific rigid body musculoskeletal computer model was developed. This model predicted muscle activation patterns, muscle forces, and angular kinematics and joint moments during walking.

**Results:**

Gait kinematics determined from motion capture matched those predicted by the model, verifying model accuracy. Primary muscles involved in generating joint moments during stance and swing were predicted by the model: at mid-stance the adductor magnus et brevis (peak activation 53.2%, peak force 64.7 N) extended the hip, and stifle flexor muscles (biceps femoris tibial and calcaneal portions) flexed the stifle. Countering vertical ground reaction forces, the iliopsoas (peak activation 37.9%, peak force 68.7 N) stabilized the hip in mid-stance, while the biceps femoris patellar portion stabilized the stifle in mid-stance and the plantar flexors (gastrocnemius and flexor digitorum muscles) stabilized the tarsal joint during early stance. Transitioning to swing, the iliopsoas, rectus femoris and tensor fascia lata flexed the hip, while in late swing the adductor magnus et brevis impeded further flexion as biceps femoris tibial and calcaneal portions stabilized the stifle for ground contact.

**Conclusion:**

The musculoskeletal computer model accurately replicated experimental canine angular kinematics associated with gait and was used to predict muscle activation patterns and forces. Thus, musculoskeletal modeling allows for quantification of measures such as muscle forces that are difficult or impossible to measure *in vivo*.

## Introduction

Chondrodystrophic dwarf breeds such as the Dachshund, Basset Hound and English Bulldog typically have shorter limbs in proportion to spine length compared to non-chondrodystrophic breeds due to disturbed endochondral ossification (Smolders et al., 2013). Therefore chondrodystrophic dwarf breeds may have distinctly different functional biomechanics and gait pathologies. Dachshunds, for example, are a common chondrodystrophic dwarf breed with high susceptibility for intervertebral disk herniation (Levine et al., 2011); pelvic sway has been shown to increase in the horizontal and vertical directions in ambulatory Dachshunds recovering from intervertebral disk herniation hemilaminectomy surgery (Sutton et al., 2016). Furthermore, although muscle activation patterns in large breed dogs have been directly measured using electromyography (EMG) during activities in a few studies (Tokuriki, 1973a, b, 1974; Wentink, 1976, 1977; Schilling et al., 2009; Deban et al., 2012), muscle activation patterns in chondrodystrophic breeds have not been described. An improved understanding of gait kinematics, kinetics, and muscle activation patterns in chondrodystrophic breeds may provide a foundation for evidenced-based therapeutic interventions such as rehabilitation following intervertebral disk herniation.

Musculoskeletal modeling has been used to understand underlying control of movement and biomechanics at the level of individual muscles in humans (Buchanan et al., 2004; Hamner et al., 2010). These models combine muscle mechanics and bone geometries with measured motion capture data and ground reaction forces to approximate biomechanical variables that are difficult or impossible to measure, such as joint moments or muscle forces. Analyzing these additional measures allows for a deeper understanding of normal and abnormal gait, as well as the recovery process after injury. In dogs, musculoskeletal modeling has been used to analyze stifle biomechanics in large breed dogs (Brown et al., 2013, 2014, 2015), and sit-to-stand biomechanics in Greyhounds (Ellis et al., 2018), but to date, functional activity in the chondrodystrophic canine population has not been investigated through modeling.

The purpose of this study is to develop a dynamic rigid body musculoskeletal model of the pelvis and pelvic limbs in a healthy chondrodystrophic dog evaluated with kinematics and kinetics during gait, and to describe pelvic limb muscle activation patterns and forces during gait. We hypothesized that pelvic limb model-predicted kinematics will not differ from experimentally measured kinematics during gait.

## Materials and Methods

### Canine Subject

This study was approved by the Texas A&M University IACUC (AUP #2013-150). A 2 year old female Dachshund (5.4 kg) with no evidence of neurologic or orthopedic disease was recruited for this study. Owner consent was obtained. The canine subject had one full body computed tomography (CT) scan, and gait kinematics and kinetics were recorded during a single session.

### Kinematic and Kinetic Data Collection

Spherical markers (9 mm diameter) were placed on the pelvis and both pelvic limbs using double sided tape. Marker locations were chosen to maximize visibility while reducing skin movement and included the cranial dorsal iliac spine, ischiatic tuberosity, greater trochanter, lateral femoral condyle, fibular head, tibial crest, lateral malleolus, calcaneus, distal 5th metatarsal, and dorsal paw. The dog’s short hair allowed effective adherence of markers. Ten walking trials were recorded using a motion capture tracking system equipped with 10 infrared cameras sampling at 100 Hz (4 MX T160, Vicon, Centennial, CO, United States; 6 MX T40-S, Vicon, Centennial, CO, United States). Additionally, sagittal plane motion was captured using digital videography (2 Bonita 720c, Vicon, Centennial, CO, United States; 100 Hz). The subject was leash walked at a slow walking pace across two symmetric and adjacent force platforms positioned level with the floor with ample distance before and after the force platforms to maintain a consistent walking speed. A full gait cycle including stance and swing was identified from each trial and defined as the duration for which the subject made contact with the left hind paw and the force platform until successive ground contact with the same paw. Recorded marker locations were processed by first smoothing using a low-pass Butterworth filter with a cutoff frequency of 6 Hz, then by filling gaps < 5 frames due to marker obstruction with quintic splines.

Pelvic limb ground reaction forces and moments were synchronously recorded at 1000 Hz using two force platforms (OR-6-6/OR-6-7 Force Plates, AMTI Technologies, Newton, MA, United States). During each trial, the canine subject traversed the force platforms so that each pelvic limb paw contacted a separate force platform. Trials where multiple simultaneous paw contacts occurred on one force platform were discarded. A successful trial was defined as one where a pelvic limb strike could be isolated for the duration of stance and no marker gaps ≥ 5 frames were present. One gait cycle was identified from the 10 cycles collected as a representative gait cycle and used for model simulation.

### CT Scan

The canine subject was intravenously administered dexmedetomidine (125 mcg/m^2^) and positioned in dorsal recumbency. A full body CT scan (Somatom Definition AS 40 slice helical CT scanner, Siemens, Munich, Germany) was performed using a 1 mm slice thickness and 0.375 mm pixel size. Atipamezole was administered directly after the CT scan to reverse the effects of dexmedetomidine.

### Tissue Segmentation

CT images were imported into medical imaging and processing software (Mimics, Materialise, Leuven, Belgium). Pelvis and pelvic limb bone geometry, segment mass, muscle volume, joint center locations, muscle attachment points, and muscle lines of action were determined from CT images. Tissues were differentiated based on Hounsfield intensity values so that a clear distinction between cortical bone (>662), trabecular bone (661 to 226), muscle (225 to −69), and fat (−70 to −205) was observed. A mask was created for each tissue which stored the locations of pixels for each threshold range. Individual bone and muscle masks were refined by manually tracing observable boundaries in each CT slice based on a comprehensive guide of canine skeletal and muscle anatomy (Evans and deLahunta, 2004).

### Pelvis and Pelvic Limb Segments

Nine bony segments were defined: the pelvis and bilateral femur, tibia, tarsus, and phalanges. Bone axes for each segment were defined based on canine and human studies which conformed to guidelines specified by the International Society of Biomechanics (Grood and Suntay, 1983; Wu et al., 2002; Fu et al., 2010). Origins and segment coordinate systems were established (Table 1). Model flexion-extension joint angles were determined based on three clinical axes in each segment using joint coordinate systems (Grood and Suntay, 1983; Fu et al., 2010; Brown et al., 2018). Flexion-extension was defined as rotation of the distal segment about a unit vector along the proximal segment z-axis.

**TABLE 1:** Canine model anatomical coordinate systems and directional unit vector axes.

Coordinate system	Origin	X axis	Y axis	Z axis
Pelvis	COM	$\vec{x}=\frac{{\vec{V}}_{R\,I\,C},-,{\vec{V}}_{R\,I\,T}}{\left|{\vec{V}}_{R\,I\,C}-{\vec{V}}_{R\,I\,T}\right|}$	$\vec{y}=\frac{\vec{x}\times \left({\vec{V}}_{R\,I\,C}-{\vec{V}}_{L\,I\,C}\right)}{\left|\vec{x}\times \left({\vec{V}}_{R\,I\,C}-{\vec{V}}_{L\,I\,C}\right)\right|}$	$\vec{Z}\;=\;\vec{x}\;\times \;\vec{y}$
Right femur	CFH	$\vec{x}=\frac{\vec{y}\times \left({\vec{V}}_{L\,F\,C}-{\vec{V}}_{M\,F\,C}\right)}{\left|\vec{y}\times \left({\vec{V}}_{L\,F\,C}-{\vec{V}}_{M\,F\,C}\right)\right|}$	$\vec{y}=\frac{{\vec{V}}_{C\,F\,H},-,{\vec{V}}_{M\,F\,C}}{\left|{\vec{V}}_{C\,F\,H}-{\vec{V}}_{M\,F\,C}\right|}$	$\vec{Z}\;=\;\vec{x}\;\times \;\vec{y}$
Right tibia	MPTC	$\vec{x}=\frac{\vec{y}\times \left({\vec{V}}_{L\,M}-{\vec{V}}_{M\,M}\right)}{\left|\vec{y}\times \left({\vec{V}}_{L\,M}-{\vec{V}}_{M\,M}\right)\right|}$	$\vec{y}=\frac{{\vec{V}}_{M\,P\,T\,C},-,{\vec{V}}_{M\,P\,M}}{\left|{\vec{V}}_{M\,P\,T\,C}-{\vec{V}}_{M\,P\,M}\right|}$	$\vec{Z}\;=\;\vec{x}\;\times \;\vec{y}$
Right tarsus	MPM	$\vec{x}=\frac{\vec{y}\times \left({\vec{V}}_{5\,M\,T}-{\vec{V}}_{2\,M\,T}\right)}{\left|\vec{y}\times \left({\vec{V}}_{5\,M\,T}-{\vec{V}}_{2\,M\,T}\right)\right|}$	$\vec{y}=\frac{{\vec{V}}_{M\,P\,25\,P},-,{\vec{V}}_{M\,P\,25\,D}}{\left|{\vec{V}}_{M\,P\,25\,P}-{\vec{V}}_{M\,P\,25\,D}\right|}$	$\vec{Z}\;=\;\vec{x}\;\times \;\vec{y}$
Right phalanges	MP25D	$\vec{x}\;=\;\vec{y}\;\times \;\vec{z}$	$\vec{y}=\frac{\left({\vec{V}}_{M\,P\,25\,D}-{\vec{V}}_{P\,C\,O\,M}\right)\times \vec{z}}{\left|\left({\vec{V}}_{M\,P\,25\,D}-{\vec{V}}_{P\,C\,O\,M}\right)\times \vec{z}\right|}$	$\vec{z}=\frac{{\vec{V}}_{5\,M\,T},-,{\vec{V}}_{2\,M\,T}}{\left|{\vec{V}}_{5\,M\,T}-{\vec{V}}_{2\,M\,T}\right|}$
Left femur	CFH	$\vec{x}=\frac{\vec{y}\times \left({\vec{V}}_{M\,F\,C}-{\vec{V}}_{L\,F\,C}\right)}{\left|\vec{y}\times \left({\vec{V}}_{M\,F\,C}-{\vec{V}}_{L\,F\,C}\right)\right|}$	$\vec{y}=\frac{{\vec{V}}_{C\,F\,H},-,{\vec{V}}_{M\,F\,C}}{\left|{\vec{V}}_{C\,F\,H}-{\vec{V}}_{M\,F\,C}\right|}$	$\vec{Z}\;=\;\vec{x}\;\times \;\vec{y}$
Left tibia	MPTC	$\vec{x}=\frac{\vec{y}\times \left({\vec{V}}_{M\,M}-{\vec{V}}_{L\,M}\right)}{\left|\vec{y}\times \left({\vec{V}}_{M\,M}-{\vec{V}}_{L\,M}\right)\right|}$	$\vec{y}=\frac{{\vec{V}}_{M\,P\,T\,C},-,{\vec{V}}_{M\,P\,M}}{\left|{\vec{V}}_{M\,P\,T\,C}-{\vec{V}}_{M\,P\,M}\right|}$	$\vec{Z}\;=\;\vec{x}\;\times \;\vec{y}$
Left tarsus	MPM	$\vec{x}=\frac{\vec{y}\times \left({\vec{V}}_{2\,M\,T}-{\vec{V}}_{5\,M\,T}\right)}{\left|\vec{y}\times \left({\vec{V}}_{2\,M\,T}-{\vec{V}}_{5\,M\,T}\right)\right|}$	$\vec{y}=\frac{{\vec{V}}_{M\,P\,25\,P},-,{\vec{V}}_{M\,P\,25\,D}}{\left|{\vec{V}}_{M\,P\,25\,P}-{\vec{V}}_{M\,P\,25\,D}\right|}$	$\vec{Z}\;=\;\vec{x}\;\times \;\vec{y}$
Left phalanges	MPMT	$\vec{x}\;=\;\vec{y}\;\times \;\vec{z}$	$\vec{y}=\frac{\left({\vec{V}}_{M\,P\,25\,D}-{\vec{V}}_{P\,C\,O\,M}\right)\times \vec{z}}{\left|\left({\vec{V}}_{M\,P\,25\,D}-{\vec{V}}_{P\,C\,O\,M}\right)\times \vec{z}\right|}$	$\vec{z}=\frac{{\vec{V}}_{2\,M\,T},-,{\vec{V}}_{5\,M\,T}}{\left|{\vec{V}}_{2\,M\,T}-{\vec{V}}_{5\,M\,T}\right|}$

*V, vector; COM, center of mass; RIC, right iliac crest; RIT, right ischiatic tuberosity; LIC, left iliac crest; CFH, center of femoral head; LFC, lateral femoral condyle; MFC, medial femoral condyle; LM, lateral malleolus; MM, medial malleolus; MPTC, midpoint of medial and lateral tibial condyles; MPM, midpoint of medial and lateral malleoli; 5MT, 5th metatarsal; 2MT, 2nd metatarsal; MP25P, midpoint of 2nd and 5th proximal metatarsals; MP25D, midpoint of 2nd and 5th distal metatarsals; PCOM, phalanges center of mass.*

### Segment Inertial Properties

Volume of cortical bone, trabecular bone, muscle, and fat was determined for each segment. Mass for each segment was calculated using

(1) $$m=\sum_{i=1}^{4}\rho_{i}\,P^{2}\,S\,N$$

where i =  1,2,3,4 refers to cortical bone, trabecular bone, muscle, and fat, ρ is the respective tissue density, P is the pixel width and height (0.325 mm), S is the slice thickness (1 mm), and N is the total number of pixels. Average densities for cortical bone (2.003 g/cm^3^), trabecular bone (1.911 g/cm^3^), muscle (1.06 g/cm^3^), and fat (0.95 g/cm^3^) were based on available canine data (Gong et al., 1964; Ragetly et al., 2008). Inertial parameters were defined about anatomical axes and relative to the center of mass (COM) of each segment using methods previously described (Amit et al., 2009).

### Pelvic Limb Joints

The center of rotation for the hip joint was defined as the center of a sphere fitted to the surface of the acetabulum (Wu et al., 2002). The hip joint had 3 degrees of freedom (flexion/extension, adduction/abduction, and internal rotation/external rotation). The stifle joint was represented as a sliding hinge with three degrees of freedom (flexion/extension, cranial/caudal translation, and ventral/dorsal translation where femorotibial translations were a function of flexion/extension). Femorotibial contact was maintained without interpenetration throughout the full range of flexion/extension. The stifle joint center of rotation was positioned at the tibia origin. The tarsal joint was a hinge with one degree of freedom (flexion/extension). The tarsal joint center of rotation was positioned at the center of the contact between the distal tibia and proximal talus. The phalanges were fixed relative to the tarsus.

### Pelvic Limb Muscles

Muscle origin and insertion points were defined based on CT data and a canine anatomical atlas (Evans and deLahunta, 2004). Muscles included in the model are in Table 2. A muscle line of action was defined for each muscle that connected origin and insertion and passed through the center of discrete cross sections perpendicular to the long axis of each muscle. Additionally, points on the centerline were added if necessary to describe anatomical changes in direction or wrapping around bone. Muscle volume was determined based on number of pixels in all CT image slices for each segmented muscle multiplied by slice thickness. Pennation angle (α) for each muscle was determined using the average of values obtained from anatomic studies of dogs (Shahar and Milgram, 2001; Williams et al., 2008; Ellis et al., 2018).

**TABLE 2:** Model input data: pelvic limb muscle volumes, pennation angles, optimal fiber lengths, tendon slack lengths, and maximum isometric forces derived from CT data and the literature.

Muscle	Volume (cm^3^)	Pennation angle (°)	Optimal fiber	Tendon slack	Maximum isometric
			length (cm)	length (cm)	force (N)
Adductor longus	1.7	41	2.2	0.7	13.3
Adductor magnus et brevis	35.1	4	3.9	0.5	202.4
Biceps femoris (patellar)	13.1	0	5.1	5.5	57.5
Biceps femoris (tibial)	13.1	0	4.7	5.0	62.5
Biceps femoris (calcaneal)	13.1	0	6.5	6.9	45.5
Extensor digitorum lateralis	0.3	10	0.4	10.0	17.7
Extensor digitorum longus	4.2	2	2.5	9.2	38.0
Flexor digitorum profundus	5.7	33	1.5	11.6	71.7
Flexor digitorum superficialis	3.5	30	1.6	16.8	42.6
Gastrocnemius lateralis	6.7	17	2.5	6.5	57.6
Gastrocnemius medialis	5.0	21	2.1	6.6	50.0
Gemelli	1.0	1	0.7	1.0	30.3
Gluteus medius	26.2	13	3.3	1.3	175.6
Gluteus profundus	2.4	3	2.9	0.5	18.9
Gluteus superficialis	4.7	13	4.4	0.6	23.5
Gracilis	9.4	5	2.8	5.8	76.6
Iliopsoas	16.9	6	2.7	2.0	140.0
Obturator externus	2.0	1	2.2	0.1	20.7
Obturator internus	3.5	0	3.3	1.3	23.9
Pectineus	1.3	8	0.9	2.5	31.5
Peroneus longus	1.0	10	0.7	6.9	30.2
Popliteus	1.1	0	2.0	0.4	12.4
Quadratus femoris	1.8	13	2.3	0.5	28.9
Rectus femoris	10.7	11	3.7	6.8	17.3
Sartorius caudalis	1.8	2	8.9	2.5	4.6
Sartorius cranialis	4.8	3	11.3	0.8	9.5
Semimembranosus (tibial)	17.0	0	7.0	0.4	54.5
Semimembranosus (femoral)	9.2	0	5.8	0.3	35.6
Semitendinosus	15.6	0	6.3	2.8	55.7
Tensor fascia lata	6.2	12	4.1	6.0	33.5
Tibialis cranialis	3.5	13	2.7	4.0	28.1
Vastus lateralis and intermedius	19.2	10	4.3	4.6	99.0
Vastus medialis	8.2	13	4.1	5.2	43.7

Muscle optimal fiber lengths were determined based on anatomic and morphometric studies of canine pelvic limbs (Shahar and Milgram, 2001; Williams et al., 2008; Ellis et al., 2018) using the following scaling factor

(2) $$\frac{f}{f_{r\,e\,f}}=\frac{l}{l_{r\,e\,f}}$$

where f is the subject optimal fiber length, f_*r*__*ef*_ is the average optimal fiber length obtained from the scientific literature, l is the length of the primary bony segment associated with the muscle, and l_*r*__*ef*_ is the average length of the corresponding bony segment measured in the scientific literature. Tendon slack lengths were defined using

(3) $$\frac{T\,S\,L}{T\,S\,L_{r\,e\,f}}=\frac{l}{l_{r\,e\,f}}$$

where TSL is the subject tendon slack length and TSL_*ref*_ is the average tendon slack length obtained from the scientific literature. Muscle tendon unit length (MTU, distance of muscle line of action from origin to insertion through intermediate points) was determined for each muscle in the model at mid-stance. Muscle optimal fiber length and tendon slack length were then adjusted using

(4) $$f_{a\,d\,j}=\left(M\,T\,U-f-T\,S\,L\right)\,\frac{f}{f+T\,S\,L}+f$$

and

(5) $$T\,S\,L_{a\,d\,j}=\left(M\,T\,U-f-T\,S\,L\right)\,\frac{T\,S\,L}{f+T\,S\,L}+T\,S\,L$$

where f_*adj*_ and TSL_*adj*_ are the adjusted muscle optimal fiber and tendon slack lengths, respectively.

Muscle optimal fiber length and tendon slack lengths were manually adjusted so that normalized fiber length (ratio of fiber length during simulation to optimal fiber length) was between 0.8 and 1.2 during mid stance and between 0.5 and 1.5 throughout the entire gait cycle (Ellis et al., 2018). Maximum isometric force (F_*iso*_) for each muscle was determined using

(6) $$F_{i\,s\,o}=T\,\left(\frac{V\,o\,l\,\sin\alpha }{f_{a\,d\,j}}\right)$$

where Vol is muscle volume and T is muscle specific tension (Knarr et al., 2013). Specific tension was estimated as 22.5 N/cm^2^ based on mammalian muscle characteristics (Spector et al., 1980; Brown et al., 1982; Roy et al., 1982; Powell et al., 1984).

### Computer Model Assembly

A pelvis and bilateral pelvic limb musculoskeletal model was developed using OpenSim (OpenSim, Stanford University, Stanford, CA, United States), an open source biomechanical rigid body modeling platform (Delp et al., 2007; Seth et al., 2018). Pelvic limb bones, joints, and muscles were based on data obtained from CT imaging. Additionally, virtual markers were placed on the model segments in locations corresponding to experimental marker positions. To represent caudal abdominal mass that is unsupported by the thoracic limbs, a torso segment representing the caudal abdominal mass (1.8 kg) was attached to the pelvis segment equal to 1/2 of the total dog mass (1/2 of 5.4 kg) minus the mass of all other model segments (0.9 kg). This assumes that the overall canine body mass is equally distributed on the forelimbs and hind limbs. The torso segment COM was located midway between the iliac crest markers, midway between the midpoint of the lumbosacral and T1 markers, and at the vertical height of the acetabulum. Mass of body regions cranial to the caudal abdomen supported by the thoracic limbs was not included in the model.

Model-predicted limb kinematics were determined for a representative gait cycle including stance and swing by minimizing the least-squares error between measured and virtual marker trajectories during dynamic walking trials (Delp et al., 2007). Marker tracking weights were adjusted to minimize errors determined during Inverse Kinematics analysis. Model-predicted kinematic outcomes included hip, stifle, and tarsal joint flexion/extension during the stance and swing portions of the gait cycle. A Residual Reduction Algorithm was used to adjust both model COM and kinematics, so as to reduce dynamic inconsistencies between ground reaction forces and kinematics. Residual actuators were placed at the pelvis COM and represented linear and rotational actuation necessary to maintain model dynamic consistency with measured kinematics and ground reactions. Residual actuators are typically needed in modeling to balance residual forces and moments that occur due to aggregate differences between experimental and model estimations (e.g., differences between experimental and model marker locations). Reserve actuators were placed at each joint degree of freedom to achieve experimental kinematics during residual reduction. Kinematics resulting from the Inverse Kinematics process were filtered at 6 Hz using a low-pass Butterworth filter during residual reduction, and residual actuator contributions were minimized. Finally, joint moments and forces determined from residual reduction were subdivided into individual muscle forces using Static Optimization which minimizes the sum of squared muscle activation across all muscles while including muscle force-length-velocity dynamics (Anderson and Pandy, 2001). Muscle activation levels were determined throughout the gait cycle, and joint moments and velocities were used to calculate joint power.

### Kinematic Analysis

Motion capture data were reconstructed using motion analysis software (Nexus 2.1.1, Vicon, Centennial, CO, United States), and marker trajectories were analyzed using a custom macro (Excel, Microsoft, Redmond, WA, United States). Pelvic limb sagittal plane kinematics (flexion-extension) were determined for the hip, stifle, and tarsal joints for each gait cycle. A three dimensional coordinate system was defined for the pelvis, and proximal-distal axes were defined for the femur, tibia, and tarsus. The pelvis coordinate system axes were oriented the same as described for the computer model. The femoral proximal-distal axis (y direction) was the vector from the lateral femoral condyle to the greater trochanter, the tibial proximal-distal axis (y direction) was the vector from the lateral malleolus to the lateral femoral condyle, and the tarsal proximal-distal axis (y direction) was the vector from the 5th metatarsal to the calcaneus. Hip flexion (α) was defined as the angle formed between the pelvis x (cranial-caudal) axis and the femoral y (proximal-distal) axis using

(7) α=c⁢o⁢s-1⁢(xp⇀p⋅yf⇀f||xp⇀p||⋅||yf⇀f||)

where x_*p*_ is the pelvis x-axis and y_*f*_ is the femur y-axis. Stifle flexion (β) was defined as the angle formed between the femoral axis and tibial axis (y_*t*_) using

(8) β=c⁢o⁢s-1⁢(yf⇀f⋅yt⇀t||yf⇀f||⋅||yt⇀t||)

and tarsus flexion (γ) was defined as

(9) γ=c⁢o⁢s-1⁢(yt⇀t⋅yt⁢r⇀t⁢r||yt⇀t||⋅||yt⁢r⇀t⁢r||)

which is the angle formed between the tibial axis and the tarsal axis (y_*tr*_). Kinematic outcomes were visually compared to assure general agreement using synched sagittal plane video.

Flexion-extension determined from motion capture data was compared to flexion-extension determined using the computer model following residual reduction. Comparison between the model-predicted kinematics and motion capture experimental kinematics was conducted using a correlation coefficient (r), defined as

(10) $$r=\frac{\sum_{i=1}^{n}\left(f_{i}-\bar{f}\right)\,\left(g_{i}-\bar{g}\right)}{\sqrt{\sum_{i=1}^{n}{\left(f_{i}-\bar{f}\right)}^{2}\,\sum_{i=1}^{n}{\left(g_{i}-\bar{g}\right)}^{2}}}$$

and standard deviation of residuals (σ_*e*_) defined as

(11) $$\sigma_{e}=\sqrt{\frac{n\,\sum_{i=1}^{n}{\left(f_{i}-g_{i}\right)}^{2}-{\left[\sum_{i=1}^{n}\left(f_{i}-g_{i}\right)\right]}^{2}}{n\,\left(n-1\right)}}$$

where f_*i*_ represents model-predicted outcomes at each time point and g_*i*_ represents the mean at each time point across all (10) experimental trials for each outcome. $\bar{f}$ is the model average value across all time points in a gait cycle, and $\bar{g}$ is the experimental average value across all time points in a gait cycle. n is the number of data points (100). Validity range was established at r ≥ 0.80 and σ_*e*_ ≤ 20% of the peak experimental value (Johansson et al., 2009).

## Results

### Musculoskeletal Computer Model

**FIGURE 1:**
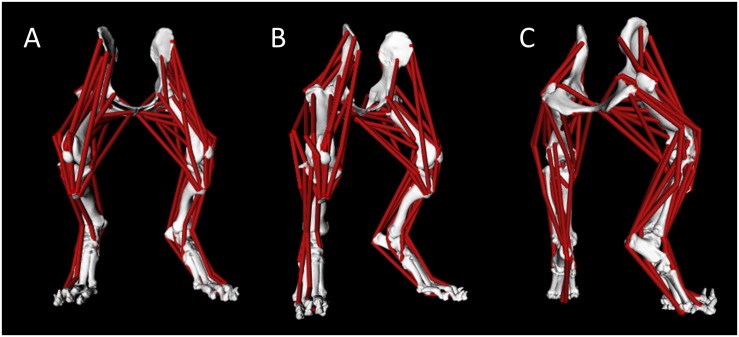
Canine pelvis and bilateral pelvic limb rigid body musculoskeletal computer model with muscle lines of action from cranial view **(A)**, right-oblique cranial view **(B)**, and right-oblique caudal view **(C)**.

A pelvis and bilateral pelvic limb rigid body musculoskeletal computer model was developed (Figure 1). Model muscle parameters for the pelvic limb are listed in Table 2.

### Kinematics and Kinetics

**FIGURE 2:**
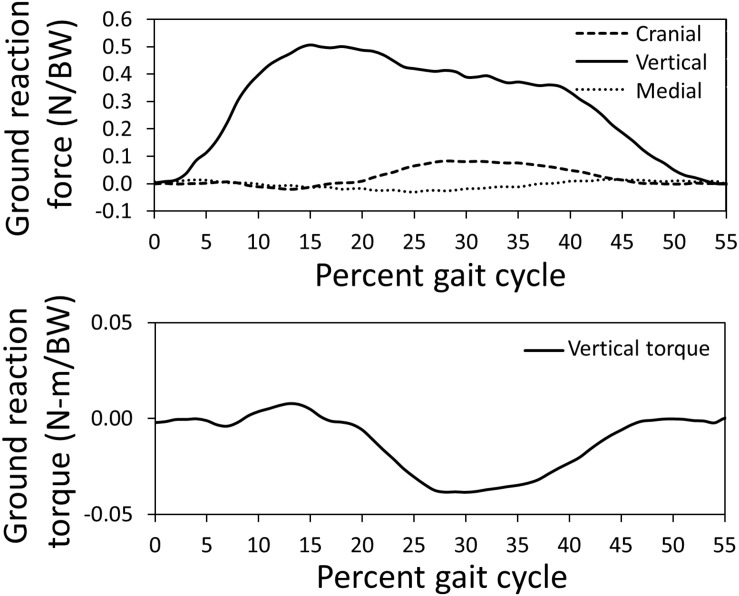
Pelvic limb ground reaction forces and torque about vertical measured using a force platform normalized by body weight (BW) for the gait cycle used for model simulation. The stance portion of gait is shown.

The canine subject walked at a slow pace of 0.6 m/s for the representative trial used to simulate gait using the computer model; the computer model gait speed (0.6 m/s) during simulation was similar. Average dog speed for the trials in this study was 0.7 ± 0.1 m/s. Measured ground reaction forces and the vertical torque from a representative trial applied to the left limb of the model are indicated in Figure 2. Pelvic limb ground reactions demonstrated the negative cranial force corresponding to braking that occurs during peak vertical force, followed by a positive cranially directed force associated with propulsion. Similarly, the slightly positive vertical torque which would resist any limb internal rotation during braking, was followed by slightly negative vertical torque which would offer resistance to any external rotation during propulsion.

**FIGURE 3:**
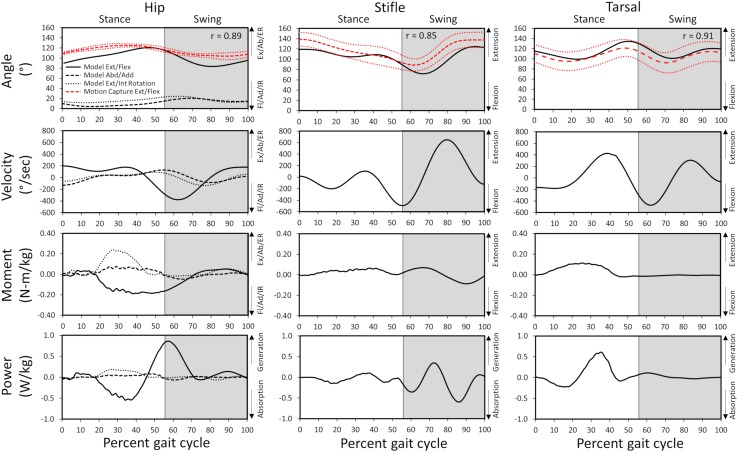
Model-predicted and experimental motion capture pelvic limb hip, stifle, and tarsal joint angles (°) and model-predicted joint angular velocities, reaction moments, and power during gait. The stance portion of gait has a white background while the swing portion of gait has a gray background. Correlation coefficients between the model-predicted and experimental flexion/extension angles are included for each joint. Experimental joint angles represent the average for 10 trials (red dashed line) with ± 1 standard deviation (dotted red line). Ex, extension; Fl, flexion; Ab, abduction; Ad, adduction; ER, external rotation; IR, internal rotation.

Gait kinematics determined from experimental motion capture (average kinematics across 10 gait cycles) were compared with those predicted by the model (single gait cycle) to verify model accuracy. Correlation coefficients of 0.89, 0.85, and 0.91 for the hip, stifle, and tarsal joint angles, respectively, and standard deviation of residuals of 6.3 (where 20% of peak experimental value =  25.0), 8.9 (where 20% of peak experimental value =  22.4), and 4.4 (where 20% of peak experimental value =  29.9) for the hip, stifle, and tarsal joint angles, respectively, show that model-predicted stance and swing kinematics determined from residual reduction algorithm were similar to experimental motion capture data (Figure 3). Model-predicted stance and swing joint angular velocities also are reported in Figure 3.

### Joint and Muscle Dynamics

**FIGURE 4:**
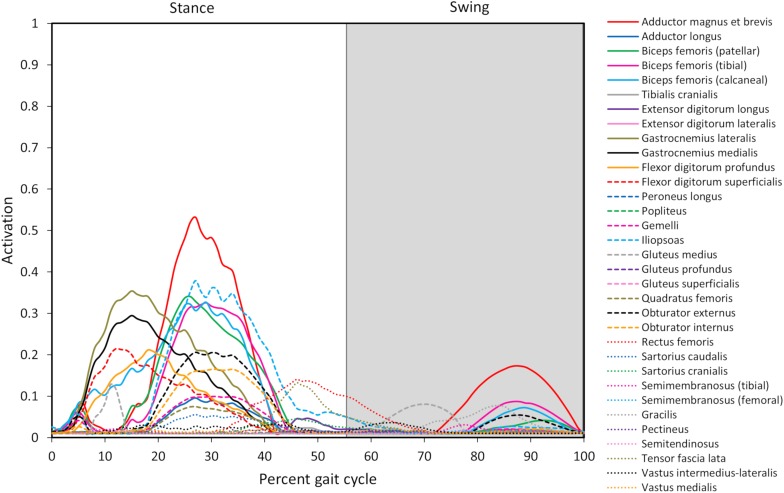
Model-predicted pelvic limb muscle activations throughout the gait cycle. Activation ranges from 0 (0% activation) to 1.0 (100% activation). The stance portion of gait has a white background while the swing portion of gait has a gray background. The adductor magnus et brevis (red line) has the highest level of activation during stance and swing.

**FIGURE 5:**
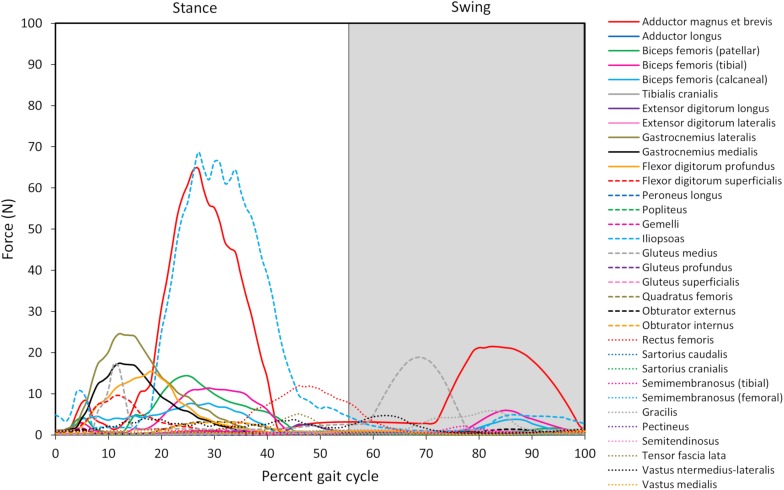
Model-predicted pelvic limb muscle forces throughout the gait cycle. The stance portion of gait has a white background while the swing portion of gait has a gray background. The adductor magnus et brevis (red line) and iliopsoas (light blue dashed line) generate the greatest force during stance, and the adductor magnus et brevis generates the greatest force during swing.

Joint reaction moments and power (Figure 3), muscle activations (Figure 4), and muscle forces (Figure 5) were determined throughout the gait cycle using the developed model. Peak activation and the phase of gait at which it occurred for each muscle were also determined (Table 3).

**TABLE 3:** Model-predicted pelvic limb peak muscle activations and forces and the timing during gait at which peaks occurred.

Muscle	Peak activation	Timing of peak activation	Peak force (N)	Timing of peak force
	(% of cycle)	(% of cycle)		(% of cycle)
Adductor longus	9.6	27	0.8	27
Adductor magnus et brevis	53.2	27	64.7	27
Biceps femoris (patellar)	34.1	26	14.4	25
Biceps femoris (tibial)	32.5	29	11.4	29
Biceps femoris (calcaneal)	32.7	29	7.7	29
Extensor digitorum lateralis	1.7	49	0.4	46
Extensor digitorum longus	4.7	48	2.6	46
Flexor digitorum profundus	21.1	18	15.5	18
Flexor digitorum superficialis	21.4	12	9.6	12
Gastrocnemius lateralis	35.4	15	24.6	12
Gastrocnemius medialis	29.5	15	17.4	12
Gemelli	9.9	31	1.6	5
Gluteus medius	12.8	11	18.9	69
Gluteus profundus	1.7	11	0.5	60
Gluteus superficialis	1.8	11	0.4	70
Gracilis	7.8	84	5.9	82
Iliopsoas	37.9	27	68.7	27
Obturator externus	20.6	27	3.2	31
Obturator internus	16.5	31	3.1	34
Pectineus	2.2	41	0.8	34
Peroneus longus	1.7	49	0.5	83
Popliteus	1.3	49	0.2	86
Quadratus femoris	7.5	27	0.7	26
Rectus femoris	14.1	46	12.0	46
Sartorius caudalis	5.5	27	0.4	27
Sartorius cranialis	4.6	46	0.5	45
Semimembranosus (tibial)	3.1	78	2.1	77
Semimembranosus (femoral)	1.6	13	0.6	76
Semitendinosus	2.1	8	1.6	78
Tensor fascia lata	13.2	46	5.1	46
Tibialis cranialis	2.4	48	0.9	46
Vastus lateralis and intermedius	3.6	64	4.7	62
Vastus medialis	2.0	65	1.1	60

#### Overall Model-Predicted Muscle Activations During Gait

**FIGURE 6:**
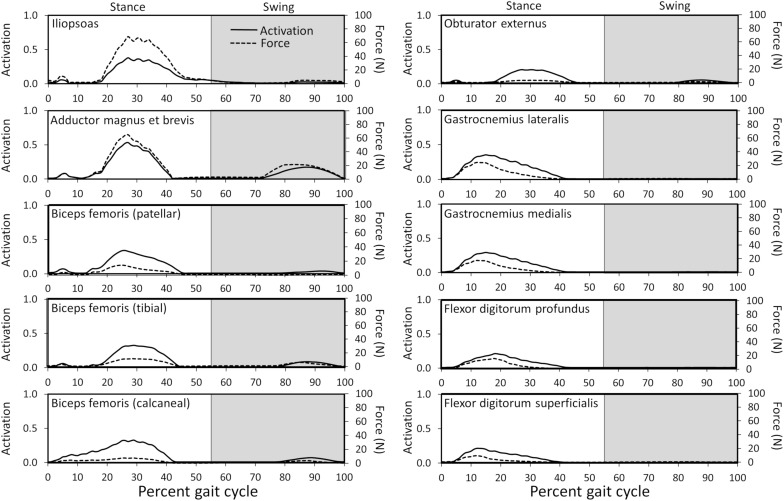
Model-predicted activations and forces throughout the gait cycle for muscles with ≥ 20% activation levels. Activation ranges from 0 (0% activation) to 1.0 (100% activation). The stance portion of gait has a white background while the swing portion of gait has a gray background.

Ten muscles including the iliopsoas, adductor magnus et brevis, biceps femoris (patellar, tibial, and calcaneal lines of action), obturator externus, gastrocnemius lateralis, gastrocnemius medialis, flexor digitorum profundus, and flexor digitorum superficialis had peak muscle activations that were ≥ 20% of maximum activation level during the gait cycle (Figure 6). Peak muscle activation of 53.2% occurred in the adductor magnus et brevis at 27% of the gait cycle corresponding to approximately mid-stance. Similarly, the iliopsoas, biceps femoris (patellar, tibial, and calcaneal lines of action), and obturator externus had peak activations occurring at approximately mid-stance. The plantar flexor muscles (gastrocnemius lateralis, gastrocnemius medialis, flexor digitorum profundus, and flexor digitorum superficialis) had peak activations occurring prior to mid-stance and remained activated throughout mid-stance. Overall, muscle activity was greater during stance (average activation across all muscles during stance =  5.1%) compared to swing (average activation across all muscles during swing =  1.8%). The adductor magnus et brevis had the highest peak activation (17.3%) during swing at 87% of the gait cycle.

#### Overall Model-Predicted Muscle Forces During Gait

Peak muscle force generally corresponded with the timing of peak muscle activation. The iliopsoas muscle had the greatest peak force (68.7 N) while the adductor magnus et brevis had the second highest peak force (64.7 N); both peak muscle forces occurred at 27% of the gait cycle (Figure 6). All other muscles generated peak muscle forces ≤ 24.6 N during stance. The adductor magnus et brevis generated the greatest force during swing (21.5 N at 82% of the gait cycle) followed by the gluteus medius (18.9 N at 69% of the gait cycle).

#### Model-Predicted Muscle Activations and Forces Associated With Joint Motion During Gait

Coordination of muscle activation during gait is critical to stability and mobility. Activated muscles generate forces and moments necessary to actuate and stabilize pelvic limb joints, and each muscle or group of muscles orchestrates a particular action during the various stages of gait. Muscle activations and forces for each joint are described below.

##### Hip joint

In early stance a slight hip extension moment was generated by the adductor magnus et brevis and gluteus medius (peak activation 12.8%) to extend the hip and produce forward movement. A sustained stabilizing hip flexion moment (Figure 3) was then generated by the iliopsoas (Figures 4, 5; peak activation 37.9%, peak force 68.7 N) to prevent limb collapse against vertical ground reaction forces when the vertical reaction force moved caudal to the hip joint at 25% of the gait cycle. At mid-stance, hip extension and external rotation began to increase (Figure 3) to facilitate propelling the pelvis forward while maintaining ground contact, and the adductor magnus et brevis (Figures 4, 5; peak activation 53.2%, peak force 64.7 N) and obturator externus (Figures 4, 5; peak activation 20.6%, peak force 3.2 N) were active. This hip external rotation moment corresponds to the peak vertical reaction torque (Figure 2; occurs 30% through the gait cycle). The flexion moment generated primarily by the iliopsoas also absorbed energy as indicated by negative hip power through mid-stance (Figure 3). In late stance hip extension changes to hip flexion and continued through early swing to bring the limb forward. In early swing the iliopsoas, rectus femoris (Figures 4, 5; peak activation 14.1%, peak force 12.0 N) and tensor fascia lata (Figures 4, 5; peak activation 13.2%, peak force 5.1 N) flexed the hip while the gluteus medius (Figure 5; peak force 18.9 N) internally rotated the hip (Figure 3). These flexor muscles, along with the adductor magnus et brevis also produced a slight adduction moment in early swing (Figure 3; −0.04 N-m) once the paw cleared the ground to bring the limb toward the sagittal mid line. In late swing the adductor magnus et brevis impeded further flexion and prepared the limb for paw strike (Figures 3–5).

##### Stifle joint

The stifle extension moment generated by the biceps femoris patellar portion from early stance through mid-stance (Figures 3–5) corresponds to absorbed energy (negative power, Figure 3) to stabilize the limb during braking while the tibial (Figures 4, 5; peak activation 32.5%, peak force 11.4 N) and calcaneal (Figures 4, 5; peak activation 32.7%, peak force 7.7 N) portions of the biceps femoris flex the stifle allowing hip extension (Figure 3). In late stance, hip flexors crossing the stifle (rectus femoris and tensor fascia lata) also extend the stifle (Figure 3) for propulsion off ground. Transitioning to early swing, the stifle flexes (gracilis, Figures 4, 5; peak activation 7.8%, peak force 5.9 N) to facilitate limb ground clearance. The patellar portion of the biceps femoris then extends the stifle (Figures 3–5) in late swing while the tibial and calcaneal portions of the biceps femoris provide antagonist activation to slow extension and prepare the limb for ground contact.

##### Tarsus joint

The plantar flexor muscles: medial gastrocnemius (peak activation 29.5%, peak force 17.4 N), lateral gastrocnemius (peak activation 35.4%, peak force 24.6 N), flexor digitorum profundus (peak activation 21.1%, peak force 15.5 N), and flexor digitorum superficialis (peak activation 21.4%, peak force 9.6 N), stabilize the tarsal joint during early stance (Figures 4, 5) as the tarsal joint flexes and energy is absorbed during ground contact (Figure 3). The tarsal joint then extends in mid stance (Figure 3) with the extension moment for propulsion generated from these muscles with additional activity from the calcaneal portion of the biceps femoris (Figures 4, 5; peak activation 32.7%, peak force 7.7 N). Tarsal extension stops in late stance (Figure 3); in late swing the biceps femoris calcaneal portion (Figures 4, 5) extends the tarsal joint in preparation of paw placement.

## Discussion

In this study, we developed the first canine bilateral pelvic limb musculoskeletal computer model of a chondrodystrophic breed to predict muscle forces and activations during gait and verified our model’s capability to predict experimental kinematics. This model provides the first step toward gaining an improved understanding of muscle activations and forces during gait. Computer models offer the advantage of predicting outcomes that are difficult or impossible to measure *in vivo* in both healthy and unhealthy dogs. Additionally, muscle activation patterns and/or functional muscle modules (Ivanenko et al., 2004; Neptune et al., 2009) determined using such models could potentially elucidate early underlying neuromuscular or orthopedic disorders or associated predispositions in certain canine breeds. Our model predicted the adductor magnus et brevis, iliopsoas, gastrocnemius, and biceps femoris muscles were the predominant active muscles during the stance phase of gait and the adductor magnus et brevis and rectus femoris muscles were the predominant active muscles during swing. During stance, the adductor magnus et brevis, iliopsoas, gastrocnemius, and biceps femoris muscles all demonstrated a moderate level of activation (>25%), which is consistent with published canine EMG gait studies (Table 4). Model-predicted outcomes showing that the adductor magnus et brevis and rectus femoris muscle activation levels were > 10% during the swing phase also are consistent with previous EMG studies of canine gait (Table 4).

**TABLE 4:** Pelvic limb muscle activations during gait compared to other studies.

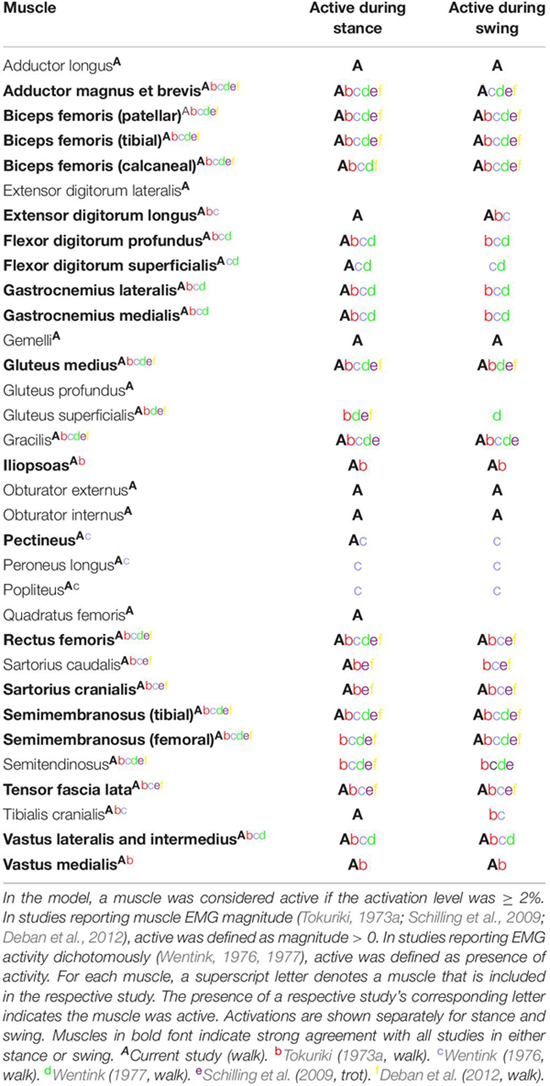

### Computer Model

The computer model developed in this study is to our knowledge the first bilateral pelvic limb canine model developed in the OpenSim platform. Our model-predicted kinematics were verified to be similar to kinematics measured *in vivo* with correlation coefficients of 0.89, 0.85, and 0.91 for hip, stifle, and tarsal joint flexion-extension, respectively, and standard deviations of residuals < 20% of peak experimental values for all joints. Furthermore, our model predicted predominate muscle activity during early and mid-stance which coincides with peak braking and weight acceptance (peak vertical ground reaction force at 16% gait cycle) and development of forward propulsion during the latter portion of mid-stance leading to paw push off (secondary peak vertical ground reaction force and peak cranial ground reaction force at 27% gait cycle), respectively. During these instances, muscles developed forces necessary to generate joint torques capable of overcoming gravitational loads to prevent limb collapse and propel the pelvic limbs forward. Less prominent muscle activation compared to stance was present in late swing to flex the extended hip and thereby prepare the limb for paw placement.

Peak muscle activation in our model was 55%, and most muscles were activated less than 38% throughout the gait cycle. Similarly, human models have predicted peak activations during walking that remain primarily below 100% activation (Thelen and Anderson, 2006; Thompson et al., 2013). Model-predicted activation levels below 100% for repetitive, low-energy daily activity such as walking provide confidence in model predictions, as maximal activations could lead to muscle fatigue and would not be expected during these activities. In contrast, a unilateral model of the Greyhound pelvic limb was previously developed to assess the sit-to-stand task. In that model some muscle activations were reported to reach maximum (100%) for sustained portions of the task (Ellis et al., 2018). Other models developed to evaluate high-energy tasks in humans such as running (Hamner et al., 2010) and jumping (Anderson and Pandy, 1999) also predicted peak muscle activations approaching or at 100%.

Use of this musculoskeletal computer model demonstrates that additional information about key outcomes such as muscle activations and forces, which are difficult or impossible to measure *in vivo*, can be gleaned non-invasively. EMG can be used to determine if a muscle is active, but extent of activation can be difficult to quantify, especially in dogs which cannot voluntarily exert maximal isometric contraction which serves as a basis for EMG signal processing. Others have used average or peak EMG signals throughout a trial to normalize muscle activity (Schilling et al., 2009; Deban et al., 2012), but the extent of activation relative to a muscle’s maximum activation potential is lost using this approach. Furthermore, surface EMG may inaccurately quantify muscle activity in individual muscles when multiple muscles are in close proximity. Fine wire electrodes may more effectively isolate individual muscles than surface electrodes but require more invasive implantation. Furthermore, the presence of EMG electrodes could potentially alter gait and affect findings. Finally, although not reported here, additional measures such as active and passive muscle fiber forces which cannot be measured *in vivo* could also be determined using the musculoskeletal model.

### Muscle Activations

#### Stance Phase of Gait

Our model predicted the adductor magnus et brevis, iliopsoas, gastrocnemius, and biceps femoris muscles as the predominant muscles active during the stance phase of gait. Muscle activation peaked at 53% in the adductor magnus et brevis. Furthermore, the iliopsoas (mid stance), gastrocnemius (early stance), and biceps femoris (mid stance) muscles demonstrated a moderate level of activation (>25%). Muscles active > 10% during stance include the flexor digitorum superficialis and profundus (early stance), obturator externus and internus (mid stance), gluteus medius (early stance), rectus femoris (late stance), and tensor fascia lata (late stance). Finally, muscles active > 5% during stance included the gemelli (mid stance), adductor longus (mid stance), quadratus femoris (mid stance), and sartorious caudalis (mid stance).

Muscle activations predicted using our computer model demonstrated similarities with measured EMG signals recorded in walking and trotting dogs (Tokuriki, 1973a, b; Wentink, 1976, 1977; Schilling et al., 2009; Deban et al., 2012) as shown in Table 4. In some studies (Tokuriki, 1973a, b; Schilling et al., 2009; Deban et al., 2012) the magnitude of activity was recorded, while in other studies (Wentink, 1976, 1977) the presence or absence of activity was recorded. In instances where agreement between our model and EMG studies was lacking, model-predicted level of activation or timing may differ. For example, the cranial and caudal semimembranosus (femoral and tibial lines of action) and semitendinosus were activated similarly in the model in terms of timing during early stance as reported by others (Tokuriki, 1973a, b; Wentink, 1976, 1977), but activation levels of the cranial semimembranosus (femoral line of action) and semitendinosus were lower (< 2%) in the computer model.

#### Swing Phase of Gait

Our model predicted the adductor magnus et brevis and rectus femoris muscles as the predominant muscles active during swing. Muscle activation was less compared to stance with peak activation of 17% in the adductor magnus et brevis in late swing. Additionally, the rectus femoris muscle was activated > 10% in early swing, while other muscles activated > 5% included the iliopsoas (early swing), tensor fascia lata (early swing), gluteus medius (mid swing), gracilis (late swing), and biceps femoris (late swing).

During the swing phase of gait, model-predicted muscle activations demonstrated similarities with measured EMG signals recorded in walking and trotting dogs (Tokuriki, 1973a, b; Wentink, 1976, 1977; Schilling et al., 2009; Deban et al., 2012) as shown in Table 4. In some cases there are inconsistencies in EMG findings across studies preventing meaningful comparisons with model predictions. As an example, some measured adductor magnus et brevis activity in late swing (Tokuriki, 1973; Wentink, 1977), Wentink reported activity only during early stance (Wentink, 1976, 1977), Deban et al. (2012) reported activation centered at the transition from swing to stance, Schilling et al. (2009) reported activation during late swing that diminished throughout stance in trotting dogs, and Tokuriki (1973) reported minimal activation throughout the gait cycle.

#### Sources of Differences in Activations Across Studies

Activation comparison was not possible in all muscles given that some muscles were not assessed in previous canine gait studies. For muscles where agreement was lacking between our model and other studies, differences can be due to a variety of reasons. Dog and breed differences could contribute to dissimilarities. Differing gait speed can lead to differing muscle activation patterns and levels (Deban et al., 2012). Additionally, our model used an optimization algorithm that minimized the maximum squared muscle stress in all muscles, but neural networks *in vivo* may recruit muscles based on other objectives such as minimizing energy consumption (Bhargava et al., 2004), and motor unit recruitment is dependent on force generation requirements (Henneman et al., 1965). For example, in the model, stifle extensor muscles active during early stance included the biceps femoris while in the study by Tokuriki (1973a) the biceps femoris, vastus lateralis, and vastus medialis were active indicating reduced synergistic muscle activation in the computer model. Differing synergistic activation between the model and EMG measured by Tokuriki could be due to differing strategies used to recruit muscles to perform tasks.

### Muscle Forces

Muscle force trends generally coincided with muscle activations (see Figure 6), and qualitative differences (e.g., the muscle with peak force not corresponding to the muscle with peak activation) are due to muscle architectural differences and contraction dynamics. For instance, the iliopsoas generated the greatest force (69 N) in our model and was active during stance but required less activation than the adductor magnus et brevis. Muscle forces during gait in the dog *in vivo* have not been measured. Some *in silico* studies have estimated pelvic limb muscle forces using optimization algorithms (Shahar and Banks-Sills, 2004; Brown et al., 2013). The biceps femoris, gracilis, and lateral gastrocnemius were reported to generate peak forces during stance in one study (Brown et al., 2013) while the vastus muscles generated the greatest force during swing in another study (Shahar and Banks-Sills, 2004). The computer model developed by Brown et al. (2013), did not include muscles acting on the femur originating from the pelvis such as the iliopsoas or adductor magnus et brevis. However, high lateral gastrocnemius force, which resists gravity, was predicted during stance in that study as well as by our model. Shahar and Banks-Sills reported the iliopsoas and adductor magnus et brevis did not produce maximum force during stance compared to other muscles, but adductor magnus et brevis force was present throughout stance (Shahar and Banks-Sills, 2004) which is in agreement with our model predictions.

### Kinematics

Model joint kinematics computed during model simulation were compared to kinematics calculated using marker trajectory data to confirm model predicted kinematics are reliable. Computer modeling processes including inverse kinematics and residual reduction may lead to kinematics that differ from kinematics determined based on measured surface marker positions. However, being that the correlation coefficients were all ≥ 0.85 for flexion-extension of each joint, the musculoskeletal computer model reliably predicted walking gait kinematics.

While model joint kinematics showed similarities to other breeds during walking gait (DeCamp et al., 1996; Hottinger et al., 1996; DeCamp, 1997; Kim et al., 2008; Fu et al., 2010; Ragetly et al., 2010), comparisons to Dachshunds could only be conducted with one study (Sutton et al., 2016). Our model demonstrated agreement with joint range of motion to trotting gait data reported for healthy Dachshunds for the hip joint during swing (36° in the model compared to 28.7°), stifle joint during stance (13° in the model compared to 13.9°), and tarsal joint during swing (40° in the model compared to 44.3°). However, range of motion in the hip joint during stance (30° in the model compared to 14.6°), stifle joint during swing (37° in the model compared to 55.9°), and tarsal joint during stance (28° in the model compared to 16.3°) demonstrated less agreement. These findings indicate more even distribution of motion across the pelvic limb joints in the model during separate stance and swing phases of walking compared to data reported by Sutton et al. (2016). However, range of motion throughout the gait cycle in Dachshunds evaluated by Sutton and colleagues is uncertain because neither minimum and maximum joint angles nor kinematic time histories are reported.

### Kinetics

Measured pelvic limb kinetics (Figure 2) demonstrated many similarities to data presented for other breeds (DeCamp, 1997; Helms et al., 2009). The vertical ground reaction force was composed of an initial peak of 51% body weight followed by a plateau region which correspond to paw placement and limb stabilization associated with braking (peak) and propulsion (plateau). Similarly, the cranial-caudal ground reaction force was composed of a caudal (braking) force for the first third of the stance phase followed by a cranial (propulsion) force for the final two thirds of the stance phase. Mediolateral forces were also lowest in magnitude. The vertical reaction torque demonstrated resistance to internal rotation during braking followed by resistance to external rotation during propulsion.

### Joint Moments and Power

Muscle activation timing and magnitude are important to generate limb movement patterns. Model-predicted pelvic limb muscle activation generated hip, stifle, and tarsal joint moments and power which resulted in dynamic walking gait. Intricacies of joint moments and power and their relationship to muscle activity and limb movement across gait sub-phases is described for each joint as follows.

A slight hip extension moment in early stance developed by the adductor magnus et brevis and gluteus medius was converted to a hip flexion moment by eccentric contraction of the iliopsoas in mid stance that continued through early swing as reported previously (Ragetly et al., 2010). A hip abduction and external rotation moment was generated by the adductor magnus et brevis and obturator externus and present during stance to abduct and externally rotate the limb. During early swing, the flexion moment generated by concentric contraction of the flexor muscles (iliopsoas, rectus femoris, and tensor fascia lata) pulled the limb forward, and in late swing the adductor magnus et brevis absorbed energy to control flexion before concentrically contracting to generate energy thereby stabilizing the limb for paw placement.

An extension moment at the stifle during stance first absorbed energy as the biceps femoris patellar portion controlled stifle flexion when the paw was placed on the ground and then extensor muscles (biceps femoris patellar portion) generated power to extend the stifle for push off (Colborne et al., 2005; Ragetly et al., 2010). In the present study, the stifle moment was predominantly extension, whereas others reported a transition from flexion moment to extension moment during stance (Colborne et al., 2005; Ragetly et al., 2010). During swing, the extension stifle moment generated by the rectus femoris and tensor fascia lata prepared the limb for paw placement prior to the flexor muscles (tibial and calcaneal portions of the biceps femoris) absorbing energy to stabilize the limb as indicated by others (Ragetly et al., 2010).

A tarsal joint extension moment during stance indicated the extensor muscles (gastrocnemius and flexor digitorum) first absorbed energy to stabilize the limb at initial weight bearing and then generated energy to provide active push off in late stance which is in agreement with other studies (Colborne et al., 2005; Ragetly et al., 2010; Headrick et al., 2014). During swing, the tarsal joint moment and power become minimal as previously indicated for Labrador Retrievers (Ragetly et al., 2010).

Joint kinetics allowed the model to replicate gait characteristics observed experimentally (Figure 3) for the dog modeled in our study.

### Limitations

These findings are encouraging given the ability to non-invasively quantify muscle activation and force during gait in a chondrodystrophic dog. Furthermore, this model could be applied to other chondrodystrophic dogs using scaling techniques to adjust model size and parameters to individual dogs. However, the following limitations should be considered with this musculoskeletal model. In this study we have modeled a chondrodystrophic breed; non-chondrodystrophic dogs could generate differing muscle activations and forces. Our model was utilized to evaluate one gait cycle from a single dog. Model development is typically done using a representative experimental event (i.e., one trial from a single dog) in order to validate the model and assure model outcomes represent the experiment. Attempting to develop a model representing multiple dogs would prevent us from validating the model given that the combined kinematics and kinetics of multiple dogs is purely hypothetical and cannot be represented experimentally. Our approach is a critical first step toward developing a generalizable model. Moreover, to assure that the model represented variations within the single canine subject, model outcomes were compared to the average of 10 experimental trials. Future steps will include comparing kinematic and kinetic outcomes from our single subject to other Dachshunds to determine generalizability. Evaluation of additional gait cycles from the same dog or additional dogs may yield kinematics and associated muscle activation and force trends not described herein. Our model demonstrated kinematic trends with general agreement to experimental data. Small differences in kinematics (e.g., hip angle) between model-predicted and experimental data could be due to slight differences in model virtual marker locations and experimentally placed markers. Additionally, model virtual markers are rigidly fixed to respective segments while experimental markers may contain motion artifact due to soft tissue and fur movement. Canine musculoskeletal modeling has inherent challenges, particularly with defining musculotendon parameters (Dries et al., 2016), but characterization of pelvic limb musculature (Shahar and Milgram, 2001; Williams et al., 2008; Ellis et al., 2018) has improved accuracy of musculotendon actuator implementation. In our model muscle characteristics such as optimal fiber length, tendon slack length, and pennation angle were estimated from values reported for larger, non-chondrodystrophic breed dogs and may differ from muscle characteristics of chondrodystrophic breeds. To maintain modeled muscle in physiologically reasonable ranges, muscle parameters were tuned so that all muscle normalized fiber lengths were between 0.8 and 1.2 during mid-stance and between 0.5 and 1.5 during the entire gait cycle (Ellis et al., 2018). For a repetitive, daily functional activity such as walking gait, we expect biomechanics would be optimal in a healthy dog and therefore anticipate that muscle characteristics would operate in ideal ranges as described. Other muscle characteristics, such as maximum isometric force, muscle line of action, and origin/insertion location were determined from CT data. Segmentation of individual muscles from CT data can be subjective when muscle boundaries are not clear. Therefore, all muscle segmentation was done in conjunction with confirmation with a canine dissection atlas (Evans and deLahunta, 2004). Muscles that originate or insert over a broad region or that do not follow a distinct line of action were defined using the following: the center of a region defined the attachment point (e.g., center of attachment on ilium for gluteus medius) or the muscle was divided into multiple lines of action (e.g., three lines of action to define the biceps femoris). Muscle wrapping around bone was approximated when necessary using via points, which are points that define a muscle’s path when within a specified joint range of motion. Muscle lines of action were visually inspected during the full range of motion in the model to confirm muscles appropriately wrapped around bony geometry. In our study, EMG was not collected during gait. Although EMG could be used to confirm model muscle activation patterns of the dog modeled in this study, use of EMG has respective limitations as described previously. Muscle activation patterns predicted using the computer model were compared to other reported canine gait studies, and agreement was present. Finally, our model included the pelvis and pelvic limbs. To improve computational efficiency of pelvic limb biomechanics and reduce model complexity, thoracic limbs were excluded.

This study investigated walking gait in a healthy Dachshund. Further investigation of other ambulatory tasks in a larger sample of dogs and in dogs with pathologies is warranted to assess gait kinematics and muscle activations in healthy dogs and dogs with neuromuscular and/or orthopedic trauma/disease to identify functional biomechanics as well as compensatory mechanisms and muscles to target for rehabilitation geared toward restoring function.

## Conclusion

A bilateral pelvic limb musculoskeletal computer model was developed for a Dachshund. The computer model accurately replicated canine gait and predicted muscle activations and forces. Musculoskeletal modeling allows for quantification of measures such as individual muscle activation and forces that are difficult or impossible to measure *in vivo*.

## Data Availability Statement

The datasets generated for this study are available on request to the corresponding author.

## Ethics Statement

The animal study was reviewed and approved by the Texas A&M University IACUC (AUP #2013-150). Written informed consent was obtained from the owners for the participation of their animals in this study.

## Author Contributions

GB, NB, and DH conceived of the project and evaluated and interpreted model results. GB, NB, DH, and GL obtained funding for the project. GB, NB, DH, GL, and JL contributed to experimental design. GB, NB, and GL provided project oversight. GL and JL recruited the canine subject and collected kinematics, kinetics, and CT data. GB, NB, and GS developed the computer model and ran simulations. GL, JL, and DH provided clinical guidance. GB and NB drafted the manuscript. All other authors reviewed, edited, and gave final approval for publication.

## Conflict of Interest

The authors declare that the research was conducted in the absence of any commercial or financial relationships that could be construed as a potential conflict of interest.
